# The Multi-faceted Effects of COVID-19 on Female Reproductive Health: An Updated Narrative Review

**DOI:** 10.7759/cureus.57944

**Published:** 2024-04-09

**Authors:** Adnan A Al-Bdairi, Hayder A Makki, Osama Shawki, Sallama H Alkhudair, Nadia M Al-Hilli, Batool A Alkhalidi, Hanan K Alkadhim, Ashwaq A Shweliyya

**Affiliations:** 1 Infertility, Assisted Reproductive Technologies, Teba IVF and Genetic Center, Babylon, IRQ; 2 Pharmacology and Toxicology, College of Pharmacy, University of Babylon, Babylon, IRQ; 3 Gynecology Surgery, School of Medicine, Cairo University, Cairo, EGY; 4 Fertility, Assisted Reproductive Technology, Alzaitoon Specialized Fertility Center, Baghdad, IRQ; 5 Obstetrics and Gynecology, College of Medicine, University of Babylon, Babylon, IRQ; 6 Obstetrics and Gynecology, College of Medicine, University of Kufa, Najaf, IRQ; 7 Obstetrics and Gynecology, Iraqi Fertility Society, College of Medicine, University of Babylon, Babylon, IRQ; 8 Obstetrics and Gynecology, Teba IVF and Genetic Center, Babylon, IRQ

**Keywords:** family planning, pregnancy complications, assisted reproductive technology (art), covid-19 vaccination, menstrual cycle, reproductive health, sars-cov-2 infection

## Abstract

Concerns about the SARS-CoV-2 pandemic's possible impact on sexual and reproductive health have grown significantly. In this narrative review, the latest research on the effects of SARS-CoV-2 infection on several facets of sexual and reproductive health is reviewed.

The review starts initially by going through the possible consequences of SARS-CoV-2 on female menstrual cycles. The virus may interfere with the menstrual cycle, which could affect hormone synthesis and endometrial reactions, according to newly available evidence. Menstrual blood loss may be impacted by COVID-19's potential to influence endothelial cell function and systemic hemostasis. Hypothalamic amenorrhea may be brought on by severe COVID-19 disease. There is little research on this subject, although most women resume their regular menstrual cycles after 1-2 months of recuperation.

The review also examines how SARS-CoV-2 infection may affect assisted reproductive technology (ART) treatments. There are few clinical data, although some research points to potential effects on embryo quality. Overall, ART results, however, did not materially change from the time before the epidemic.

Obstetric problems are more likely when SARS-CoV-2 infection occurs during pregnancy, especially in the third trimester. Even though the maternal death rate is still low, pregnant women, especially those with comorbidities, are more likely to experience serious sickness.

The review emphasizes how the COVID-19 vaccine affects menstrual cycles, showing brief, modest modifications without serious health hazards. Also included are the psychological effects of family planning choices during the pandemic.

In conclusion, this narrative review offers a thorough assessment of the complicated and changing effects of SARS-CoV-2 on sexual and reproductive health. The different requirements of people and couples during and after the pandemic are highlighted, underscoring the necessity for ongoing study and specialized healthcare practices.

## Introduction and background

In December 2019, a novel, infectious, multi-system disease caused by the severe acute respiratory syndrome coronavirus 2 (SARS-CoV-2) and coronavirus disease 2019 (COVID-19) first appeared [1]. Healthcare systems all over the world are being severely overburdened by the disease. As of December 9, 2022, it had caused 6,630,082 fatalities and 643,875,406 confirmed cases. The most common clinical signs of SARS-CoV-2 viral infection range from symptomless to moderate influenza-like sickness and lung infection (mostly fever, malaise, cough, and exhaustion), to progressive disease with concomitant lung injury, multiple organ failure, in addition to death [2-4]. Recent information emphasizes that the virus can attack additional organs and systems. Diarrhea, nausea, or vomiting are reported in only a tiny proportion of cases, indicating a possible involvement of the gastrointestinal tract [3].

Additionally, studies have revealed that SARS-CoV-2 infection or vaccines, and Long COVID-19 syndrome may affect reproductive health. These potential long-term effects are not known [5]. Even though both men and women are equally prone to getting COVID-19 [3], males are more expected to die from the illness. This has raised the possibility that female sex hormones function as a form of defense against serious illness. Sex hormone receptors are present throughout the body, expressed in almost all tissues and organs, and have functions unrelated to reproduction [6]. Both sexes with acute SARS-CoV-2 infection exhibit numerous significant clinical and immunological variations; women had lower severity of inflammation, increased lymphocyte cell counts, and quicker immune responses [3, 4]. The immunomodulatory, antiplatelet, and vasodilatory actions of estradiol are well-known. However, infection during pregnancy is linked to more serious illness. In several observational studies, menstrual alterations have been linked to both [7].

Female fertility is unaffected by the COVID-19 vaccination as documented by several researchers [8]. Menstrual cycle disturbance has been identified in women with long COVID-19 in small-scale investigations [9]. Menstrual abnormalities are prevalent in general, as is COVID-19 infection and/or immunization, but it's also crucial to keep in mind that existing observational studies may be biased. It is unclear how common menstrual alterations were, or if reported menstrual irregularities are caused by COVID-19 indirectly or by regular fluctuations in menstrual symptoms. In general, menstrual health is rarely given much attention in medical studies. This is also true in the case of SARS-CoV-2, as it was not examined in studies examining SARS-CoV-2 infection, treatment, or vaccination [5]. However, since women make up around 70% of healthcare providers and social care staff globally, these populations were more likely to be exposed to the virus. Given this, the COVID-19 era demands that female fertility and reproductive health receive greater attention.

Therefore, the effects of SARS-CoV-2 infection, pandemic, vaccine, and Long COVID-19 syndrome on female reproductive well-being are discussed in this narrative overview of the existing evidence. Data about the effects of SARS-CoV-2 infection during pregnancy, on sexual health, besides in women undergoing assisted reproductive technology (ART) are also reviewed. Given the general lack of published short and long-term data in these fields, we propose a substantial research priority for the future.

Additionally, we'll consider a practical clinical strategy for the menstrual cycle or reproductive health difficulties that can arise in this situation.

## Review

Materials and methods

A systematic approach to data collection, analysis, and presentation completed the study's methodology and ensured the validity and trustworthiness of the thorough summary of SARS-CoV-2's effects on female reproductive health. The authors used a methodical approach to explore scholarly resources for papers and research on SARS-CoV-2 and female reproductive health. Peer-reviewed studies, reviews, and meta-analyses published up until the knowledge cutoff date in January 2023 were the inclusion criteria. The exclusion criteria were research with duplicates, inadequate data, or papers published in languages other than English.

A section on menstrual health, ART, pregnancy, vaccination, long COVID, hormonal impact, and reproductive organs was also created by the writers from a subset of the literature. A cross-verification of the extracted data was conducted to guarantee accuracy and dependability. The classification was based on the major findings, statistics, and methodology that were taken from each study within the chosen categories. Research design, sample size, and statistical techniques used to evaluate the quality of each study. We assessed each study's limitations and strength of evidence, taking into account the possibility of publication bias and its effect on the interpretation of results. Finally, we combined data to present a coherent narrative on the various effects of SARS-CoV-2 on female reproductive health. We also found recurrent themes and trends within research within each category.

Before evaluating the risk of bias in individual studies, taking into account variables like sample selection and potential confounders, the research team first identified gaps in the literature and areas that needed more investigation. They then made recommendations for future research directions and methodological improvements. We make an effort to guarantee that ethical standards are followed while reporting and synthesizing research findings throughout the entire research writing process.

Impact of SARS-CoV-2 on female reproductive health

The Influence of SARS-CoV-2 on the Menstrual Cycle

Evolving evidence indicates that COVID-19 infection may affect the female menstrual cycle. The Arizona CoVHORT study found that among 127 COVID-19-positive women, 16% reported menstrual changes, most commonly irregular menstruation (60%) [10]. Women have mainly reported decreased menstrual volume and a prolonged cycle [11]. Importantly, most women returned to normal menstrual patterns within 1-2 months post-recovery. Another study found no difference in menstrual characteristics between females with severe and non-severe SARS-CoV-2 but indicated potential ovarian injury and reduced ovarian reserve in infected women [11]. The current evidence suggests a potential link between COVID-19 infection and temporary menstrual cycle changes, possibly involving reduced ovarian reserve [5].

Nevertheless, these findings are based on small, early-pandemic cross-sectional studies and require further investigation.

Mechanisms of SARS-CoV-2 Impact on Female Reproductive Health

There are several hypothesized ways through which SARS-CoV-2 affects the health of female reproductive organs, including the female hypothalamic-pituitary-gonadal (HPG) axis [12]. The virus enters cells via the angiotensin-converting enzyme 2 (ACE2) receptor, found in tissues like the ovaries and endometrium, potentially impacting hormone production and endometrial responses [13]. The virus's presence in the reproductive tract may disrupt immune function, altering vaginal blood loss. SARS-CoV-2 could also interfere with vascular endothelial function and systemic hemostasis, affecting menstrual blood loss [14, 15]. Severe COVID-19 illness may induce hypothalamic amenorrhea.

SARS-CoV-2-infected female patients occasionally exhibited aberrant sex hormone concentrations, which may have indicated ovarian suppression. The decrease in inflammation brought on by cytokine storms may be accompanied by a rise in sex hormones. In addition, modifications in sex hormone concentrations may also result from nervous system impairment [16].

The Influences of SARS-CoV-2 on Follicular Fluid

A Spanish researcher investigated 16 oocytes from two asymptomatic SARS-CoV-2-positive women who were undergoing egg retrieval. None of the examined oocytes had any SARS-CoV-2 RNA, according to the research [17]. The results of Barragan were confirmed by data from a study involving a SARSCoV-2 female who had egg recovery. In the follicular fluid, they claimed there was no SARS-CoV-2 RNA present. Wearing personal protective equipment (PPE) as they carried out the oocyte retrieval, while, the cervical block was in place to lessen virus aerosolization. The airflow was switched off in the operation theater and the room of the in vitro fertilization (IVF) laboratory, and the laboratory staff strictly enforced good laboratory practice (GLP) [18].

After COVID-19, an Argentinian investigation on the follicular fluid of all women undergoing IVF, discovered anti-SARS-CoV-2 immunoglobulin IgG (immunoglobulin G) and low levels of vascular endothelial growth factor (VEGF) and interleukin (IL)-1. All women having IVF after COVID-19 infection had IgG in their follicular fluid [19]. A change in the follicular fluid's composition, which reflects oocyte quality, can harm reproductive function [20]. Researchers also discovered that cytokines such as Vascular endothelial growth factor (VEGF), transforming growth factor-B, tumor necrosis factor (TNF), platelets derived growth factor (PDGF), and numerous interleukins, have an important role in regulating ovarian physiology, particularly folliculogenesis, and ovulation, where they help to create a milieu that supports follicle growth and selection [18].

They control a variety of processes, including oocyte maturation, follicular survival/atresia, and cellular proliferation/differentiation [21-25]. The distinctive anti-SARS-CoV2 spike protein receptor binding domain (RBD) IgG in the blood and follicular fluid of infected and immunized individuals was the subject of several researches. They discovered that patients with positive anti-SARS-CoV-2 IgG serum levels also had quantities of immunoglobulins in the follicular fluid, and this finding was comparable between the two study groups: those who were infected and those who had received the vaccine. After vaccination, these antibodies was visible in blood and follicular fluid starting on day 13 following the initial dose [18].

Menstrual Disturbances and COVID-19 Severity

For fertility and reproductive health, a normal menstrual cycle is essential, and deviations can result in issues like preeclampsia, low birth weight, and metabolic changes [20, 26]. Menstrual problems in SARS-CoV-2-infected women have included worsening premenstrual symptoms, protracted periods, and decreased menstrual volume [12, 27]. The renin-angiotensin system is fundamentally made up of ACE2, which has a role in the etiology of COVID-19. Angiotensin-(1-7) depletion, increased serum ACE2 levels, down-regulation of membrane-bound ACE2, and promotion of unopposed Ang II activity are all effects of COVID-19 infection that may be related to menstruation problems [28, 29]. The increased frequency of irregular periods, amenorrhea, and menstrual discomfort in severe COVID-19 cases may be related to obesity, metabolic syndrome, dexamethasone therapy, and aspirin use [16]. The menstrual cycle may also be affected by stress while receiving medical treatment [30, 31].

Henceforth, down-regulation of ACE 2, aberrant hormone levels, medicines, and stress may all contribute to menstruation abnormalities in SARS-CoV-2-infected women. 

Hypothalamic Amenorrhea in Severe COVID-19

During the COVID-19 outbreak, women were more prone to feeling stressed. An elevated prolactin (PRL) and malfunction of the hypothalamic-pituitary-ovarian (HPO) axis could result from a recent mental condition, such as depression, anxiety, or sleeplessness, which was reported by 36 of the 75 patients (48%) in a previous study. Some people hypothesized that female patients with COVID-19 may have ovarian harm since increased immunological or inflammatory responses and disruption of the HPO axis can both contribute to aberrant ovarian function [5, 16]. Finally, hypothalamic amenorrhea may be brought on by COVID-19 serious sickness [5]. About 30% of secondary amenorrhea in women of reproductive age is caused by hypothalamic amenorrhea (HA). It is brought on by insufficient hypothalamic gonadotrophin-releasing hormone (GnRH) secretion, which in turn causes pituitary gonadotrophin and gonadal steroid release to be unsuccessful [32].

Assisted reproductive technologies and COVID-19

Implications of COVID-19 on Assisted Reproductive Technology

Limited clinical data are available on the outcomes of Assisted Reproductive Technology (ART) procedures in individuals who have had SARS-CoV-2 infection. The virus could potentially influence ovarian, endometrial function, and sperm parameters [5]. COVID-19 can activate proinflammatory cytokines, potentially interfering with folliculogenesis [33].

An observational study on couples undergoing IVF after COVID-19 recovery showed no significant differences in IVF cycle characteristics except for a lower proportion of top-quality embryos. This suggests that systemic inflammation induced by SARS-CoV-2 during development might reduce embryo quality, leading the study authors to recommend delaying IVF for 3 months interval after recovery to account for the extent of folliculogenesis [34]. Another analysis of asymptomatic or mildly infected individuals found a slight reduction in blastocyst formation rates but no other significant differences in ART outcomes, including ovarian reserve, fertilization rates, pregnancy rates, and implantation rates [35].

Observational Studies on IVF Outcomes Post-COVID-19

It's worth noting the impact of suspending ART treatments during the initial stages of the outbreak [36]. All couples' success rates with IVF decrease with delay. Due to this, older women have a lesser likelihood of having a live birth, especially those who have a known infertility etiology. The likelihood of a spontaneous conception during a 6 to 12-month period without therapy more than offsets the impact of delay in women with unexplained infertility. Clinics considering a phased return to regular clinical services after COVID-19 should give older women priority, especially those with a known reason for infertility [37]. While SARS-CoV-2 infection may impact ART outcomes to some extent, overall, ART outcomes during the pandemic do not seem to differ significantly from the pre-pandemic period. Larger-scale epidemiological studies are needed to entirely recognize the direct consequences of SARS-CoV-2 infection on ART [38].

Impact of COVID-19 on ART Outcomes

Early research suggested potential links between severe illness in partners before natural conception and complications like preterm birth and early pregnancy loss [39]. Concerns about SARS-CoV-2's impact on reproductive function led to investigations of cell entry pathways. Data eventually supported the resumption of assisted reproductive technology (ART) with strict safeguards, including patient testing and staff protocols [40]. Studies examined markers like anti-Mullerian hormone (AMH), antral follicle count (AFC), and hormone levels in women with COVID-19 [41]. While some reports showed no significant difference, others indicated lower AMH levels and hormonal imbalances [42]. Additionally, research into ART outcomes revealed variable impacts on embryo quality and pregnancy rates following COVID-19 recovery. Some studies found no significant differences, while others suggested caution regarding early ART after COVID-19 [34]. Vaccine-induced or infection-related immunity did not seem to affect ART success [43, 44]. Further research is needed to fully understand the impact of COVID-19 on both male and female reproductive health in the context of ART.

Psychological impact on ART during the pandemic

Likely, women waiting for their first infertility consultation or receiving infertility treatment may have had mental symptoms of stress, anxiety, and depression due to the COVID-19 epidemic and the suspension of assisted reproductive treatments (ART) [45]. Infertility diagnoses and therapies with ambiguous outcomes are frequently perceived as a burden. Couples who are unable to conceive are at risk for psychosocial issues, particularly anxiety and sadness. Women are more vulnerable than men to these symptoms, which seem to be more prevalent than in the general population [46].

According to a Chinese study, serum AMH levels in COVID-19 patients were significantly lower than those in healthy women [47]. Low serum levels of AMH have also been connected to psychological stress and anxiety intensity. Additionally, low plasma AMH measures were also related to emotional stress and the severity of anxiety [48]. Therefore, psychological counseling should always be offered especially during this difficult period of the pandemic.

SARS-CoV-2 infection throughout pregnancy

Prevalence and Risk Factors of SARS-CoV-2 Infection in Pregnant Women

There is no documented higher rate of SARS-CoV-2 infection in pregnant women than in the general population., with most pregnant women being asymptomatic [49]. In contrast, SARS-CoV-2-positive pregnant women are more prone to experience serious obstetric complications; their infants may be born prematurely or with low arterial pH, also pregnant individuals are at increased risk of severe disease, ICU hospitalization, and death [50]. Nevertheless, several comorbidities, such as obesity, diabetes, and gestational diabetes with insulin dependence, raise the risk of infection in pregnant women [51]. Later in pregnancy, particularly in the third trimester, COVID-19 infection is more prevalent [52]. Pregnant women have an increased chance of developing severe illnesses, frequently necessitating intensive care and mechanical breathing, especially in the third trimester. Despite the low risk of maternal mortality, COVID-19-related respiratory or thrombotic illness is the main cause of death (2.4 per 100,000) [5].

A body mass index over 25 kg/m2, being unvaccinated, diabetes, hypertension, maternal age over 35, low socioeconomic level, and racial or ethnic minorities are risk factors for serious infection [53]. Surprisingly, almost 98% of pregnant women who needed to be hospitalized for severe COVID-19 were unvaccinated.

Obstetric Complications and Maternal Outcomes

The COVID-19 pandemic has led to various obstetric complications in pregnant women, such as pre-eclampsia, caesarean sections, premature rupture of membranes, fetal distress, and preterm delivery [54]. The prevalence of these complications has been highly variable, likely influenced by the severity of the maternal infection and data sources, primarily medical records. While the data on miscarriage and stillbirths have been insufficient, termination of pregnancy has shown an increase during the pandemic due to fears of fetal infection [55].

Pre-eclampsia, a noteworthy complication, has been reported with prevalence rates ranging from 16% in asymptomatic COVID-19 cases to 63% in severe cases [54, 56]. Some researchers attribute pre-eclampsia to elevated cytokines, including IL-6, which trigger inflammation [57]. Fetal distress, related to insufficient placental oxygen supply, has been mentioned in a few reviews, with rates of 14% and 8% for fetal distress and ruptured membranes, respectively [56]. However, other studies found no significant differences between COVID and non-COVID pregnancies in terms of these complications [58].

The prevalence of caesarean sections has varied significantly, ranging from 43% to 92%, with reasons including maternal respiratory issues and excessive weight gain during the pandemic [59]. Maternal concerns about fetal infection and newborn health have also driven elective C-sections [60]. Preterm delivery rates have been lower, with some studies showing rates similar to the general population, although there have been cross-cultural differences [57, 60].

The relationship between cortisol, prenatal depression, and preterm delivery requires further investigation, alongside the evolving evidence regarding the vertical transmission of COVID-19 [61].

Vertical Transmission and Neonatal Outcomes

Numerous studies have examined the effects of COVID-19 on pregnancy, reporting a range of concerns, from little morbidity to significant morbidity. Vertical transmission is a controversial topic, and the World Health Organisation (WHO) and the Royal College of Obstetricians & Gynecologists (RCOG) both accepted that vertical transmission is possible [62].

Furthermore, SARS-CoV-2 was not found in samples of amniotic fluid, cord blood, or neonatal throat swabs in six cases [63], which provides no evidence of vertical transmission. The likelihood of negative outcomes, like spontaneous preterm birth, was not increased by COVID-19 throughout pregnancy, according to a larger study involving 99 SARS-CoV-2-infected pregnant women. The 100 infants born to these moms did not have any SARS-CoV-2 infections [64]. However, there remains a debate about the danger of vertical transmission during pregnancy due to the sparse and contradictory results from antibody and nucleic acid-based testing in newborns delivered to mothers with SARS-CoV-2.

Vertical transmission should have been cited as a major consideration by the European Society of Human Reproduction and Embryology's (ESHRE's) COVID-19 Working Group in their recommendations [40]. Vertical transmission is rare [65], and no apparent link exists between COVID-19 infection and congenital anomalies [66]. Still, there is an increased risk of stillbirth, possibly due to widespread placental changes from placental infection [67], which may also lead to fetal growth restriction. There is nearly double the risk of low birth weight [68] and symptomatic SARS-CoV-2 is associated with a 2-3 fold higher preterm birth rate, primarily due to obstetric intervention, and an increased risk of cesarean section [5].

Effects of COVID-19 Vaccination on Reproductive Health

Influence of COVID-19 Vaccines on Menstrual Cycles

Regarding COVID-19 vaccination, studies suggest minor and temporary menstrual changes. Medical staff should inform females that these menstrual changes may include longer cycles and heavier bleeding but reassure them that such disruptions are short-lived, lasting 1-2 cycles, and pose less risk to general health than SARS-CoV-2 infection, particularly in reproductive-aged females. No evidence that vaccine-related misinformation should not promote hesitancy [8].

Limited international studies on SARS-CoV-2, have examined the link between the vaccination and menstrual cycles [10, 11]. Current data proposes that COVID-19 vaccination can mildly and temporarily affect the menstrual cycle, although the exact cause remains unclear.

The largest study conducted so far, tracking menstrual information from nearly 20,000 females using the 'Natural Cycles' mobile phone application (Natural Cycles USA Corp, New York, USA), found that while the length of menses remained unaffected, the first and second vaccine doses were associated with an average increase in menstrual length of 56 and 71 days, respectively. Women receiving both vaccination dosages in the same menstrual cycle experienced a more substantial rise of 3.70 days. The HPG axis may be influenced differently during various menstrual cycle phases due to the immunological response triggered by the mRNA inoculation [69].

A Norseman study using online forms revealed that after both vaccine doses, females described heavier than customary bleeding and a raised risk of other menstrual cycle disturbances [70]. Further research is needed to understand the immunologic influences on menses in this context.

Potential Mechanisms Behind Vaccination and Menstrual Changes

This research supports the facts provided in widely circulated social media reports and adds to the conversation because there is currently little evidence on the subject. Despite preliminary data linking the COVID-19 vaccine to menstrual irregularities, medical professionals commonly see women of reproductive age who have alterations in their menstrual cycle [71]. 

Currently, two biologic routes have been proposed to explain the immunological stimulation of the menstrual cycle brought on by vaccination. First, cells of innate immunity may momentarily restrict the hormones that regulate reproduction, leading to extended cycling [72]. To substantiate this, one study found that immunization given through the follicular phase of the cycle resulted in immune cell-mediated endocrine alterations that lengthened the follicular phase [73]. When women are given hormonal contraceptives that contain estrogen and progesterone, which have been found to disrupt the body's normal hormonal cycle, this process is further reinforced [72]. The second process includes the disruption of tissue regrowth and breakdown in the uterus by natural killer cell and macrophage activity [73]. Throughout the menstrual cycle, these immunity cells regulate uterine tissue deterioration and repair [73]. This theory is supported by the link between menstrual loss and age. Given that immune systems are linked to menstrual flow, this raises the possibility that immune cells may have contributed to the observed increase in menstrual flow in older women because their endometrial lining's less effective repair mechanisms involving natural killer cells and macrophages cause higher menstrual flow rates [71]. Both a longer cycle and a larger menstrual volume may be caused by these two mechanisms when combined [73].

Influence of COVID-19 Vaccination on Ovarian Reserves

Public health authorities continue to place a high focus on comprehending the COVID-19 vaccine's effects, including those on women's ovarian reserves. To evaluate ovarian reserve, it is advised to combine the sonographic antral follicle count (AFC) and AMH values [41, 74]. The ovarian developing follicles produce AMH, which is correlated with the active ovarian pool. A sonographic assessment of the bilateral ovaries during the follicular phase of the menstrual cycle is known as the antral follicle count [20]. Previous research showed that after vaccination, AMH levels were unchanged [75].

In line with earlier findings [28], a current study comparing female ovarian reserve before and after COVID-19 vaccination reveals that the vaccine is not linked to changes in ovarian reserve by various biomarker assays of AMH and AFC [76]. It is critically need to conduct additional studies with a larger sample size to better understand the possible impacts of COVID-19 illness on female gonadal function. 

Influence of COVID-19 Vaccination on Fertility

The literature on vaccination and women's fertility was able to be found in two studies. In the initial research, 44 female rats were intramuscularly administered the full human dose of the Pfizer vaccine on days 21, 14, and nine and twenty of gestation (the end of breastfeeding), respectively, to prepare them for mating [8]. The embryo, fetus, fetus-to-be, neonate, fertility, and mating efficiency were all unaffected. The conclusion of lactation saw typical postnatal development, including growth, physical development, and brain capabilities. The Pfizer vaccination, according to the authors, was not harmful to women's ability to conceive, carry children, or give birth to them. In overall summary, to a certain extent, vaccinations might not been linked to infertility.

In a similar manner, a meta-analyses included 29 studies found no adverse effects of the COVID-19 vaccination on both males and females fertility [8]. The groups that were vaccinated and those that were not showed no differences in the biochemical and clinical pregnancy rate. The biochemical pregnancy rates between vaccinated and non-vaccinated individuals, the testosterone, FSH, and LH levels before and after Gam-COVID-Vac (also known as Sputnik V) vaccinations, and the sperm volumes before and after BNT162b2 vaccinations did not differ significantly, according to subgroup meta-analyses based on the type of vaccine.

Influence of COVID-19 Vaccination on Pregnancy

Despite the fact that at least one in-depth evaluation of the value of vaccinations and the creation of particular vaccines for expectant mothers was published in 2020, the COVID-19 vaccines were not mentioned [77]. The authors of another recent review have underlined that the placenta is thought to be the route via which the immune system of the pregnant lady travels. To give the newborn passive immunity, IgG antibodies have been deliberately delivered through the placenta [56]. IgG antibodies are the only ones that are believed to cross the placenta; as was previously mentioned, IgA antibodies are said to be unable to do so.

No research on the effects of vaccines on pregnant women could be located in the COVID literature, despite the fact that pregnant women have been mentioned as a priority for vaccination. Women who are pregnant should be included in vaccine trials, according to at least one research team. According to the authors, "First, these vaccines contain mRNA that is transported into host cells in the form of a lipid nanoparticle. The host cells in the body produce coronavirus spike proteins that elicit an immune response against SARS-CoV-2. In the local lymph nodes, this action is active. Since there is no scientific evidence to suggest that this process differs during pregnancy, we anticipate that both pregnant and non-pregnant people will benefit equally from it. Second, these vaccines don't have any adjuvants or live viruses that might harm a developing fetus. Furthermore, the information that is currently known on the Moderna mRNA vaccine's developmental and reproductive toxicity in rats has not revealed any safety concerns with regard to female reproduction, fetal or embryonic development, or postnatal development" [78]. It is interesting in light of these suggestions that information on the effects of current vaccines on pregnant women and their fetuses could not be located, and ongoing surveillance and reporting demonstrate the safety and efficacy of COVID-19 vaccines in pregnancy.

Impact of Long COVID-19 on female reproductive health

Menstrual Disturbances and Long COVID

Although the effects of protracted COVID on reproductive health in females are still poorly understood, some data may point to a connection between lengthy COVID symptoms and menstruation. In a global survey of women of reproductive age with extended COVID-19 [1792 participants), 36% of respondents had menstrual problems, 26% had irregular cycles, and 20% had heavy menses. Women who have brief disruptions in their menstrual cycles as a result of psychological stress associated with the epidemic are unlikely to suffer long-term effects on their reproductive health. However, women who experience recurrent cycle irregularities without a clear medical cause should get baseline blood work and a psychological health evaluation, with treatment based on the symptoms that are most common and local recommendations. Additionally, lifestyle counsel regarding alcohol consumption, food, exercise, and weight should be given [5].

Overlapping Symptoms of Menopause During Long COVID-19

Long COVID typically affects premenopausal women and is more common in women than in men [9, 79]. Transgender people are also disproportionately impacted. In contrast to the 3% of women aged 40 to 49 who claimed early menopause, 5% of women beyond the age of 49 had postmenopausal bleeding [79]. These observational studies do not prove a causal relationship, though.

Long COVID and menopausal symptoms like fatigue, sleep issues, cognitive fog, and palpitations share a lot of similarities. A previous study found that the majority of females ascribed their persistent SARS-CoV-2 symptoms to menopause or vice versa, which may have prevented them from taking advantage of menopausal hormone therapy to treat their symptoms [14, 69]. To learn more about the connections between extended COVID and female reproductive hormones, a longitudinal study is required.

Management Strategies for Persistent Cycle Disturbances

A woman should seek medical treatment if her menstrual cycle continues to vary after receiving a vaccination for about three months since these variations could be a sign of more serious reproductive health issues. Females may complain of pre-existing conditions exacerbated by the pandemic, such as endometriosis or Polycystic Ovary Syndrome (PCOS). A thorough assessment for common endocrinal and gynecological disorders, including PCOS and endometriosis, as well as perimenopause, is recommended. This evaluation should include weight, hormonal contraception changes, and relevant blood tests [5].

Significant concerns have been raised about the impact of extended COVID-19 on cycle disruptions, especially as many females reported chronic problems. Therefore, general management approaches for enduring cycle disturbances can be stated in the points below. Rapid diagnosis for newly-onset COVID-19 infection by the diagnostic standards is in line with the National Institute for Health and Care Excellence (NICE) recommendations [80]. Since COVID-19 has no preventative medications, the population's defense against coming into contact with the pathogen needs to be increased. The fundamentals of general treatment approaches are in agreement with the recommendations made by international health organizations, which call for rest, supportive care, maintenance of water-electrolyte balance and homeostasis, oxygen therapy, support for the respiratory system, and supportive care of the corresponding vital organs for critically ill patients.

A complete blood count is to rule out the anemia from menorrhagia. Testing for clotting problems is only advised for individuals with a lengthy history of recurrent menstrual cycle irregularities. Hormone tests are not necessary in women who experience heavy but regular menstrual loss. It is necessary to do investigations, which may include a pregnancy test, sexually transmitted infection (STI) testing, a cervical cytology test, and other workup for ongoing cycle irregularities [81-83].

The levonorgestrel-releasing intrauterine system (IUS) is still the preferred hormonal therapy for heavy cycle problems [80, 82], achieving an 80-90% decrease in menstrual bleeding, with lower treatment cost and complication risk [81, 82]. Following the elimination of contraindications, combined hormonal contraception. Continuous therapy or a long cycle of medroxyprogesterone acetate (21 days followed by a 7-day respite) [83].

Tranexamic acid plus any non-steroidal anti-inflammatory medicine may be considered non-hormonal. Finally, endometrial ablation may be a part of the surgical-non-reversible protocol. Provide short-term care while awaiting specialist care for women with fibroids that are 3 cm or more in diameter to conduct further investigations and talk about treatment alternatives [80-83].

Effects of COVID-19 on hormones and ovarian reserve

Hormonal Impact of COVID-19

The female ovarian endocrine system is only slightly negatively affected by COVID-19, according to a study. Researchers found no detectable differences in sex hormone concentrations between COVID-19 patients and controls, and monthly variations did not affect sex hormone levels [16].

At present, there is no clear indication of a sex-specific overexpression of viral entry receptors [84]. Higher estradiol was related to a higher possibility of death. In men, higher testosterone might be protecting against any considered outcome. High estradiol level was related to an increased likelihood of death in both sexes [85].

The risk of COVID-19 may be increased by low serum levels of low Testosterone/Luteinizing Hormone (LH) (T/LH), follicular stimulating hormone (FSH)/LH, and sex hormone-binding globulin (SHBG), as well as high levels of LH and Estrogen/Testosterone (E2/T). Additionally, the likelihood of elevated LH and E2/T serum levels and decreased T/LH, FSH/LH, and SHBG levels increases with COVID-19 clinical severity. Clinicians should be aware that COVID-19 might have negative effects on gonad functions [85].

However, other studies revealed that COVID-19 patients had elevated levels of hormones like FSH, LH, and PRL, which would have indicated that their ovarian function had been suppressed in response to the inflammation [86]. LH and PRL levels may have increased as a result of the nervous system-related effects of SARS-CoV-2. Hormonal levels may be impacted by mental health issues during the epidemic. The COVID-19 virus does not affect AMH, a measure of ovarian reserve [30]. More investigation is required [16].

Influence of COVID-19 on Ovarian Reserve

Previous research has shown that COVID-19 disease is followed by alterations in menstruation. The possible effects of SARS-CoV-2 on female reproductive structures, specifically the ovary, have not been adequately studied. Based on blood LH, FSH, E2, and AMH measures from menstrual cycle days 2 to 5, a recent study found that SARS-CoV-2 disease or the inflammatory response to the virus itself did not appear to alter gonadotropins and ovarian endocrine secretions in infertile females. It is vitally need to do additional research with more patients to better recognize the possible implications of SARS-CoV-2 illness on female ovarian function [75].

However, according to some researchers, women with COVID-19 can exhibit ovarian damage, including decreased ovarian reserve and a reproductive endocrine disease. The findings suggested that there may be a short-term decrease in ovarian reserve and reproductive capacity. Direct viral infection, an inflammatory or immunological response that is out of control, and failure of the HPO axis are all possible causes of aberrant ovarian function under COVID-19, which ultimately results in ovarian harm [87]. Others, on the other hand, claimed that the endocrine values were consistent with the ovarian status when the differences according to the study groups we analyzed, and divided the participants into two sets, along with their preceding AMH levels: normal-high responders (AMH>1 ng/ml) or low responders (AMH < 1 ng/ml). They concluded that having the disease has no bearing on the condition of the ovarian reserve, but that the grade of variance in AMH values is dependent on whether the female was a high or low responder [88].

Effects of COVID-19 on Reproductive Organs and Gametes

Presence of SARS-CoV-2 in Reproductive System

The material that is now accessible is unclear about SARS-CoV-2's presence in the reproductive system and its possible interaction with reproductive system physiology [89]. Gametes may be frozen as a precaution, but SARS-CoV-2 testing is advised as a must for infertile patients undertaking fertility preservation operations [90]. Whether SARS-CoV-2 is present in semen is a topic of debate [91]. While other investigations indicate conflicting results, certain studies raise the possibility of viral shedding into seminal plasma [92]. To preserve fertility, sperm cryopreservation is advocated, with a focus on utilizing extremely secure technologies to avoid possible cross-contamination [92].

Men with COVID-19-induced fever are thus advised to postpone reproductive procedures and keep an eye on sperm parameters [93]. There are also worries about the potential effects of modification on gametes and embryos [89]. The female reproductive system, in contrast, appears to be less affected. No virus presence has been identified in female gametes or reproductive tissues [94], presumably as a result of protective barriers such as the zona pellucida [95].

Risks and Cross-Contamination in Cryobanking Services

Even though there is little chance of cross-contamination between SARS-CoV-2-infected and uninfected samples during cryopreservation, concerns are nevertheless advised. To prevent potential virus contamination of biomaterials during vitrification and storage, high-security closed devices are advised [96]. These precautions include screening both spouses for SARS-CoV-2, putting hygienic cryostorage methods in place, and making sure gametes or embryos are cleaned thoroughly before [97].

In light of the COVID-19 pandemic, it is imperative to manage infertility patients and ensure the safe handling and cryopreservation of biomaterials during fertility preservation [96, 97].

Influence of COVID-19 on Assisted Reproduction

The epidemic of COVID-19 forced a reevaluation of the necessity of reproductive therapies, leading to a complicated environment formed by developing research and medical advice. The American Society for Reproductive Medicine (ASRM) initially recommended that only infertile patients who were already undergoing emergency fertility preservation finish their therapies, with a focus on embryo cryopreservation [89]. In a similar vein, despite the paucity of data on COVID-19-related pregnancies, the ESHRE advised infertile patients to postpone pregnancy by oocyte or embryo cryopreservation [98].

Influence of COVID-19 on Pregnancy Outcomes and Neonatal Status

Vulnerability of Embryos to SARS-CoV-2

The levels of co-expression of ACE2/Transmembrane protease serine 2 (TMPRSS2) and Basigin/Cathepsin L (BSG/CTSL**) **vary, according to studies on embryo cell entrance receptors. While TMPRSS2 expression begins later in blastocysts, primarily in the trophectoderm, ACE2 first occurs in embryos early [99]. Strong ACE2/TMPRSS2 co-expression in day 6 peri-implantation embryos indicates susceptibility [100]. Day 5-7 blastocysts containing ACE2/BSG are indicative of SARS-CoV-2 susceptibility. The infection of trophectoderm cells by pseudotyped virus particles demonstrates the viral entrance via the S protein-ACE2 pathway. Blastocysts are infected by live SARSCoV-2, although this is avoidable with neutralizing antibodies. Infection is more prevalent in fully hatched blastocysts and is protected by the zona pellucida, having cytopathic effects [101].

Impact of COVID-19 on Pregnancy Outcomes

There have been few meta-analyses involving pregnant COVID-19 patients, and it is still unclear how the condition and pregnancy outcomes are related. Pregnant women with COVID-19 did not appear to have increased rates of gestational diabetes, hypertensive disorders of pregnancy, or pre-eclampsia when compared to pregnant women without the virus, according to the subsequent case series [102]. Only four instances of spontaneous miscarriage or termination were recorded out of the 295 cases, with a quarter of them still undeliverable at the time of reporting. [103]. 

According to a systematic analysis, pregnant women were at higher risk for severe COVID-19 infection than non-pregnant women, but not for SARS-CoV-2 infection or symptoms of COVID-19. However, according to a different systematic review and meta-analysis about COVID-19 in pregnancy, SARS-COV2 is more likely to cause preeclampsia, stillbirth, and premature birth than COVID-19 is. When compared to COVID-19 which is asymptomatic, symptomatic COVID-19 was linked to a higher risk of caesarean delivery and premature birth [104]. Nonetheless, according to a different systematic review and meta-analysis about COVID-19 in pregnancy, SARS-COV2 is more likely to cause preeclampsia, stillbirth, and premature birth than COVID-19 is. When compared to COVID-19 that is asymptomatic, symptomatic COVID-19 was linked to a higher risk of caesarean delivery and premature birth. Severe COVID-19 was significantly linked with preeclampsia, gestational diabetes, preterm birth, and low birth weight compared to mild COVID-19 [105]. Contrary to individuals who do not have COVID-19, pregnant women who have it are more likely to birth their babies early and run a higher risk of both maternal death and ICU admission. The likelihood of their infants being admitted to the neonatal unit is higher [104].

Influence of COVID-19 on Neonatal Outcomes

Based on a consecutive series among 219 post-COVID-19 delivery cases, with gestational age at delivery ranging from 28 to 41 weeks. Neonatal Apgar scores at 1 and 5 minutes were generally favorable (7 to 10). Few newborns had low birth weight (< 2.5 kg), but approximately 1/3rd was shifted to the neonatal intensive care unit primarily for maternal infection-related investigation and monitoring. A single case of neonatal asphyxia and death was reported. Laboratory test results for 19 neonates showed only a few with increased leukocyte count and C-reactive protein (CRP), and no cases of lymphocytopenia or thrombocytopenia. Nucleic acid testing in various neonatal samples, except for three neonatal throat swabs, yielded negative results for COVID-19 [103]. However, an Iranian case series involving severe SARS-CoV-2 reported two patients of intrauterine fetal death (IUFD) that continued undelivered at the time of mother's death (of them, one involving a twin pregnancy), along with two additional neonatal deaths, also in a twin pregnancy [102].

Mechanism of infection in the female reproductive system

Relationship Between SARS-CoV-2 and ACE2

The equilibrium between angiotensin II (Ang II) and angiotensin-(1-7), two essential hormones in the renin-angiotensin system (RAS), is significantly regulated by angiotensin-converting enzyme 2 (ACE2) [29]. While Ang II causes inflammation, tissue remodeling, and vasoconstriction, Ang-(1-7) has anti-inflammatory and vasodilatory characteristics [28]. Ang-(1-9) and Ang-(1-7), respectively, are produced by the hydrolysis of Ang I and II by ACE2. The COVID-19 virus enters cells of the host via ACE2 receptors, principally through the spike (S) protein, which has a strong affinity for ACE2 [1, 4]. Transmembrane protease serine 2 (TMPRSS2) in the cytoplasm catalyzes proteolytic cleavage that facilitates this viral entrance. Infection with SARS-CoV-2 reduces ACE2 expression, which raises Ang II levels and lowers Ang-(1-7), which contributes to the inflammatory reactions found in COVID-19 patients [28, 106].

Vulnerability of embryos to SARS-CoV-2

The levels of co-expression of ACE2/TMPRSS2 and BSG/CTSL vary, according to studies on embryo cell entrance receptors. While TMPRSS2 expression begins later in blastocysts, primarily in the trophectoderm, ACE2 first occurs in embryos early [99]. Strong ACE2/TMPRSS2 co-expression in day 6 peri-implantation embryos indicates susceptibility [100]. Day 5-7 blastocysts containing ACE2/BSG are indicative of SARS-CoV-2 susceptibility. The infection of trophectoderm cells by pseudotyped virus particles demonstrates the viral entrance via the S protein-ACE2 pathway. Blastocysts are infected by live SARS-CoV-2, although this is avoidable with neutralizing antibodies. Infection is more prevalent in fully hatched blastocysts and is protected by the zona pellucida, having cytopathic effects [101].

Relationships Between ACE2 and Ovarian Function

Ovarian cells have high levels of ACE2, suggesting that they may be susceptible to SARS-CoV-2 infection during the early stages of embryo development. The equilibrium between Ang II and Ang-(1-7) is controlled by ACE2, which affects steroid secretion, follicle development, and ovulation [106]. Downregulation of ACE2 brought on by SARS-CoV-2 may impair ovarian function [5]. Age-related increases in ACE2 expression and variations in ACE2 expression across menstrual phases in the endometrium may have an impact on viral harm susceptibility [11, 56]. Endometrial diseases can result from imbalances in the ratio of Ang II to Ang-(1-7) cells. The relevance of ACE2 is shown by its function in the prognosis of endometrial cancer [28]. All RAS components are expressed by the placenta during pregnancy, and ACE2 abnormalities can result in illnesses such as preeclampsia and developmental limitation [18]. These results emphasize the importance of ACE2 in female reproductive health.

Sexual Transmission of COVID-19

Possibility of Sexual Transmission

Regarding the question of whether or not the coronavirus is transmitted through sexual contact, given the significant role that sexual activity plays in the majority of people's lives. Men and their sexual partners should receive the appropriate education regarding the seriousness of the virus transmission by semen in patients. To give the best skills to lower the risks associated with SARS-CoV-2 sexual transmission through counseling and suitable measures, healthcare practitioners need to raise their knowledge and understanding [107]. Determining infectivity, crucial for transmission, relies on factors like infectious dose and exposure route. Molecular methods largely replace virus isolation for detection but don't definitively confirm infectivity. In the context of COVID-19, [108] for up to 11 days post-hospitalization. Sexual transmission may lead to delayed outbreaks, making genital secretions' role crucial, especially for traditionally non-sexually transmitted viruses. In ART procedures, viral transmission risks, including intracytoplasmic sperm injection (ICSI), should be considered in future research [101]. 

Sexual Transmission in Women, Recommended Safety Measures, and Response of Fertility Clinics

Three researches examining female genital secretions for SARS-CoV-2 presence showed consistent results. The first studied 12 pregnant women with modest COVID-19 presentations, with negative viral vaginal smears. The second included samples from 10 females with severe SARS-CoV-2 bronchopneumonia admitted to ICUs, all of whom were negative for the viruses. The third studied 35 reproductive-age and postmenopausal patients with COVID-19 of mild to moderate severity, with all lower genital tract samples testing negative for SARSCoV-2 [109]. A systematic review of case series and case reports involving 28 pregnant women also found no SARS-CoV-2 in vaginal mucosa and breast milk. While these studies involved small patient numbers, they encompassed various age ranges and disease severities, collectively suggesting that the female genitalia are doubtful a route of COVID-19 transmission. In response to the outbreak of COVID-19, fertility organizations worldwide issued guidelines for assisted reproductive technology (ART) treatments [109]. These guidelines recommended suspending new fertility treatments, including ovulation induction, intrauterine insemination, and in-vitro fertilization, as well as postponing embryo transfers and non-urgent diagnostic measures. The aim was to mitigate potential complications from ART and pregnancy, avoid SARS-CoV-2-related complications, support healthcare resource allocation, and observe social distancing recommendations. Urgent cases, including oncology fertility preservation, were excused. Fertility clinics implemented measures like telemedicine, patient screening, and limited clinic visits during the pandemic. The recommencement of ART coincided with local epidemiological improvements and reduced healthcare resource strain. Safe reopening guidelines included virtual consultations, pre-screening, mandatory screening, patient education, staff safety measures, infection control protocols, and enhanced cleaning and disinfection. These measures ensured patient and staff safety while providing fertility care during the pandemic [40, 62].

COVID-19 transmission dynamics: sexual contact factors and changing sexual behavior with implications for contraception

Factors Affecting Infectivity of COVID-19 Through Sexual Contact

Age and gender did not significantly affect the chance of infection, but they did for the risk of serious illness. Both the risk of SARS-CoV-2 infection and severity of illness progression were impacted by co-morbid health disorders, particularly those that affected the renin-angiotensin system. The probability of severe COVID-19 was most significantly influenced by age and male sex [110].

The COVID-19 epidemic severely hampered patients' ability to get medical care, particularly STI testing. This service cutback likely led to fewer opportunities for epidemiological data collecting, laboratory testing, and diagnosis, underestimating the actual burden of STIs in 2020 [111]. IWantTheKit (IWTK, Johns Hopkins Center for Indigenous Health, Baltimore, USA), a free STI testing program that is available online and via mail, saw a considerable rise in usage during the pandemic. Due to clinic closures all through the initial pandemic wave, individuals who ordinarily sought STI testing and therapy at sexual health centers were referred to IWTK in Baltimore city [112].

Shifts in Sexual Behavior and Contraception

New sexual partnerships were prevalent in the community, were related to common other risk factors, and may aid in explaining persistent STI transmission despite the pandemic's restrictions on social gatherings. 66.2% of respondents in an online poll of COVID-19 mail-in self-collection STI test users reported having a new sexual partner. Women under 25 years old, white race in men, and higher condom use were all strongly linked to reporting a new sexual partner [111]. In addition, females had higher resistance to COVID-19 infection and illness progression due to estrogen, a better innate immune response, the ACE2 gene, and microbiome. The primary significance of related microbiota and particular environment factors in gender-based differential regarding the morbidity and mortality owing to COVID-19 in females has also been explored [113].

However, there has been a decline in the number of casual partners among "Men who have sex with men"(MSM) since COVID-19, and there may have been a brief decline in the transmission of HIV/STIs. Due to past sexual behavior and a low priority placed on avoiding COVID-19, some MSM reported casual sex partners in violation of social distancing norms. Keeping accessible HIV/STI-related testing and care available for these males during lockdowns is crucial [114]. According to a recent systematic review, COVID-19-related restrictions were associated with increased rates of sexual dysfunction and decreased sexual activity [115].

COVID-19 pandemic effects: shifting parenthood desires, psychological stress, and family planning dynamics

Changes in Parenthood Desires During the Pandemic

The COVID-19 epidemic is affecting people's desire to have children. It is uncertain whether these results will cause a significant change in the birth rate shortly [116]. Participants who had intended to have children before the pandemic abandoned the notion in a good proportion of cases due to worries about financial hardships and possible pregnancy risks in most of the cases [109]. Contrarily, during quarantine, 11.5% of individuals who had not planned to have children before the epidemic had a renewed desire for parenting, motivated by a need for a positive change in their lives (50%) and a sense of purpose (40%) but only 4.3% made an active attempt to become pregnant [116]. According to a different study, 44.6% of infertile women thought about delaying childbearing because of COVID-19 [117].

Psychological Stress and Family Planning

Stress was a major problem during the COVID-19 epidemic, as is typical during pandemics, which also tend to worsen psychological distress and symptoms of mental illness [118]. Pregnant women and infertile people may be particularly vulnerable. In terms of family planning and the desire for parenthood during a pandemic, there is a lack of capacity for social, psychological, and medical support. Future studies should also examine the psychological effects of COVID-19 on people's desires for parenthood and starting families [119, 120].

Recommendations for Infertility Patients with COVID-19

The third world countries are bearing the bulk of the burden of this battle as the weakened healthcare system struggles to halt the tide of the new coronavirus epidemic across the globe. Beginning infertility therapies, such as ART, will place additional stress on the weakened medical community at this crucial time. The future of many patients now appears to hang in the balance. It is our responsibility to focus on containing this spreading pandemic in resource-poor nations rather than on providing non-urgent treatments [121].

Everyone aged 6 months and older, including those who are expecting, nursing, attempting to conceive, or who may do so in the future, should receive the COVID-19 vaccination, according to the CDC, which also advises obtaining booster shots, if necessary. There is presently no proof that any vaccines, including COVID-19 vaccines, can lead to infertility issues in either men or women (trouble conceiving). There is presently no proof that any vaccines, including COVID-19 vaccines, can lead to infertility issues in either men or women (trouble conceiving). As further research demonstrates that the COVID-19 vaccine is safe and effective during pregnancy, the CDC and medical professionals advise COVID-19 vaccination for people who desire to have children [122].

There is no proof that COVID-19 immunizations cause fertility issues, however, results from recent research studies suggest that persons who menstruate may notice tiny, transitory alterations in menstruation after receiving the vaccine [123].

The COVID-19 vaccination has caused several women to become pregnant after receiving it, including some who received it during COVID-19 vaccine clinical trials [122]. There is presently no proof that COVID-19 vaccine components or antibodies produced after vaccination might interfere with getting pregnant either now or in the future [123,124]. Like with other vaccines, researchers continue to closely examine COVID-19 shots and will keep the world population updated as new information comes to light.

## Conclusions

SARS-CoV-2 has a broad influence on the reproductive health of women, hence targeted research is required. Menstrual problems require tailored therapy, and embryo cryopreservation is the priority when it comes to safety precautions. Parenting aspirations are changing because of the pandemic, which emphasizes the need for psychological support. Encouragement of the COVID-19 vaccine allays fears about infertility, and research is being monitored continuously.

Furthermore, the menstrual cycle may also be affected by stress while receiving medical treatment. The pandemic has introduced challenges to assisted reproductive technology treatments, and the impact on fertility and family planning decisions requires further study. Monitoring menstrual cycles post-vaccination and exploring the virus's effects on long COVID-19 symptoms are essential areas of research. Strict safeguards are necessary for assisted reproduction to address transmission concerns. Overall, this is a complex and evolving field with much more to discover.
